# Influence of BMI on the Recurrence Rate of Nephrolithiasis in the Adult Population of Saudi Arabia: A Retrospective Study

**DOI:** 10.7759/cureus.33539

**Published:** 2023-01-09

**Authors:** Mahadi B Alyami, Abdulaziz A Alshehri, Mohammed A Alzaidi, Abdullah F Asiri, Murad O Fatani, Abdulrazaq H Alahmadi, Ziyad Alnefaie, Taha A Hamoda

**Affiliations:** 1 Medicine, King Abdulaziz University Faculty of Medicine, Jeddah, SAU; 2 Urology, King Abdulaziz University Hospital, Jeddah, SAU

**Keywords:** hypertension, obesity, body mass index: bmi, recurrent nephrolithiasis, renal calculi

## Abstract

Objectives

Nephrolithiasis is a common disease, and Saudi Arabia is among the countries with the highest prevalence of nephrolithiasis. Obesity is one of the risk factors associated with the increased formation of renal calculi. We aimed to assess whether obesity also increases the recurrence rate of nephrolithiasis.

Methods

We retrospectively identified and collected data of 283 adult patients with renal stones who were managed at our hospital from November 2018 to November 2021. The demographic information, comorbidities, stone burden, and treatment methods related to them were identified, collected, and analyzed. Moreover, we performed the chi-squared test (χ2) and multivariate logistic regression analysis in order to assess the risk factors (i.e., independent predictors) of recurrence among the studied patients. Additionally, the odds ratio (OR) was calculated at a confidence interval (CI) of 95%. A *p*-value of less than 0.05 was considered statistically significant.

Results

Of the 283 adult patients we analyzed, 35 did not meet the criteria of our study and, consequently, were excluded. Therefore, we ended up with 248 patients, of whom 179 (68.1%) were males, 131 (52.8%) had a previous history of renal stones, and 90 (36.3%) had chronic illnesses. Moreover, the mean age of the studied patients was 48.91 ± 14.51 years, and the mean BMI was 29.44 ± 6.1 kg/m^2^. It was found that most of the patients (35.5%) had only one stone, while 23.4% of them had more than 5 stones. Furthermore, the majority of the stones (35.9%) were medium size (with a stone diameter of 10-19 mm [1-1.9 cm]). We did not find a relationship between obesity and the recurrence rate of renal stones. However, there was a significant association (*p* < 0.05) between the recurrence rate and Saudi nationality, chronic diseases (more specifically, HTN), unilateral stones, and a stone diameter of 10-19 mm (1-1.9 cm). Additionally, we identified diabetes and the kidney as stone location factors that are associated with less recurrence.

Conclusion

Although obesity was reported to increase the risk of renal stone formation, we did not find it to be associated with an increased recurrent rate of the disease in the Saudi Arabian population, which is inconsistent with other studies conducted in this area in other countries. Therefore, larger studies are needed to prove this finding.

## Introduction

Nephrolithiasis, also called renal stones, refers to the formation of crystal calculi in the upper urinary tract. The calculi contain calcium or phosphate minerals in addition to the organic matrix, which is composed of proteins as well as glycoproteins and are caused by the supersaturation of the minerals in urine, leading to the formation, growth, and aggregation of crystals [1]. In total, 40% of renal stones are asymptomatic, and the common symptoms reported by patients include sudden onset, colicky abdominal pain located in the flanks or anterior abdomen, urinary urgency, sweating, hematuria, nausea, and vomiting [2]. Nephrolithiasis is a common disease, with a five-year recurrence probability of 50%. Moreover, it is more common among men than women, as approximately 13% of men and 7% of women are likely to develop this disease in their lifetime [3].

Over the years, the incidence of renal stones has increased owing to factors such as the increase in obesity, diabetes mellitus, and hypertension, along with dietary changes [4]. Such an increase has had major public health implications, as the cost associated with renal stones in the United States alone increased from US$ 2 billion in 2000 to over US$ 10 billion in 2006 [1,5]. Moreover, renal stones predispose the patient to chronic kidney disease (CKD), end-stage renal disease (ESRD), hypertension, and metabolic bone disease (MBD) [6,7], all of which lead to further devastating sequelae such as the need for renal replacement therapy and an increased risk of osteoporosis and fractures.

Furthermore, renal stones demonstrate demographic, geographic, and seasonal variations, with countries that have hot, dry climates (such as Saudi Arabia) having higher prevalence. Besides, more cases are reported during the summer months [8,9]. A prospective study involving 4827 patients and a 46-year-long follow-up period concluded that obesity and weight gain increase the risk of renal stones, and the effect is greater in women than in men [10]. The overall prevalence of obesity in Saudi Arabia has been estimated to have increased from around 12% in 1992 to 41% by 2022 among men and from 21% to 78% among women [11].

Additionally, a Saudi cross-sectional study involving 173 patients, which was conducted to examine the relationship between obesity and renal stones, concluded that not only does obesity increase the risk of developing renal stones, but obese individuals also tend to have larger stones than their non-obese counterparts [12]. The lifestyle of obese individuals, centered around increased consumption of salty foods, fructose-rich beverages, and purine-rich animal proteins, may partially explain this mechanism. These have also been shown to induce urine supersaturation and changes in urine pH, which, subsequently, leads to renal stone formation [13,14].

Since there is a clear link between obesity and renal stone formation, our study aimed to investigate whether obesity also affects the recurrent rate of the disease. Some previous studies found a link between weight gain and an increased risk of renal stone formation, while others demonstrated the impact of obesity on the size of renal stones. Therefore, by investigating the link between obesity and the recurrence rate of renal stones, weight reduction programs may turn out to be a neglected aspect of health management that can optimize both the quality of life of the patients and the associated cost of renal stones on public health. Thus, this study aimed to assess the relationship between obesity and the recurrence rate of renal stones.

## Materials and methods

Study design, setting, and time

After receiving ethical approval from the Research Ethics Committee at our institution (Reference No 143-22), we conducted a retrospective study at King Abdulaziz University Hospital (KAUH). The data were collected between December 2021 and September 2022.

Study participants

The inclusion criteria were patients who were admitted to KAUH from November 2018 to November 2021 with symptomatic nephrolithiasis confirmed by radiological imaging. The patients were adults (i.e., over the age of 18), and both sexes as well as different races were included in the study. However, the patients with conditions that severely increase the formation of renal stones such as cystinuria (an inborn error of metabolism) and renal tubular acidosis were not included in the study. Additionally, the study participants with missing data were also excluded. Initially, there were 283 participants in the study, out of which 35 were excluded, which left 248 patients for the analysis.

Data collection

A retrospective chart review of the hospital records of the included patients (N = 248) was performed, and an electronic data collection form was employed to gather information concerning the patient’s height, weight, demographics, comorbidities, complications, diagnostic modalities, stone burden (i.e., number, size, location, and bilaterality), and management were collected. The BMI was calculated based on a patient’s last episode of renal stones, and recurrence was defined as having a previous history of symptomatic renal stones preceding the last episode. It should be noted that stone composition is not frequently performed at our institute; therefore, it was excluded from the study.

Data analysis

The data were analyzed using SPSS version 26. In order to test the relationship between variables, the qualitative data were expressed as numbers and percentages and the chi-squared test (χ^2^) was employed. On the other hand, the quantitative data were expressed as mean and standard deviation (mean ± SD), and the non-parametric variables were tested using the Mann-Whitney U test. Moreover, a multivariate logistic regression analysis was conducted to assess the risk factors (i.e., independent predictors) of recurrence among the studied patients. Additionally, the odds ratio (OR) was calculated at a confidence interval (CI) of 95%, and a p-value of less than 0.05 was considered statistically significant.

## Results

This study included 248 patients, whose mean age was 48.91 ± 14.51 years and mean BMI was 29.44 ± 6.1 kg/m^2^. Out of these patients, 68.1% were males, 66.9% were Saudi nationals, and 77.4% were from Jeddah city. Additionally, only 0.8% of the patients had a first/second-degree relative who was diagnosed with renal stones. About 36% (36.3%) of them reported having chronic diseases, with DM and HTN (24.6%) being the most common (Table 1).

**Table 1:** Distribution of the studied patients according to their demographics, family history of renal stones, and chronic diseases (no. 248) BMI: body mass index; DM: diabetes mellitus; HTN: hypertension; CVD: cardiovascular disease; UTI: Urinary tract infection; GIT: gastrointestinal tract

Variables	No. (%)
Age (years)	48.91 ± 14.51
BMI (kg/m^2^)	29.44 ± 6.1
Gender:	
Female	79 (31.9)
Male	179 (68.1)
Nationality:	
None-Saudi	82 (33.1)
Saudi	166 (66.9)
Residency:	
Jeddah	192 (77.4)
Outside Jeddah	56 (22.6)
First/second-degree relative diagnosed with kidney stones:	
No	246 (99.2)
Yes	2 (0.8)
If yes, state how many family members (one/two/three/more):	
One	2 (0.8)
Chronic diseases:	
No	158 (63.7)
Yes	90 (36.3)
If yes, specify:	
DM	61 (24.6)
HTN	61 (24.6)
CVD	5 (2)
Recurrent UTI	5 (2)
GIT disorders	5 (2)

Table 2 demonstrates that the most common imaging modality used to confirm the diagnosis was the non-contrast-enhanced CT (NCCT) scan (96%), and 77% underwent ureteroscopic/laser fragmentation as a form of surgical management. As regards renal stones, the most common location was the kidney (42.3%), and for those having stones in the kidney or ureter, 63.3% of the stones were unilateral. Moreover, most of the patients (35.5%) had only one stone, while 23.4% of them had more than five stones. Besides, the majority (35.9%) of them had a stone diameter of 10-19 mm (1-1.9 cm).

**Table 2:** Distribution of the studied patients according to clinical data related to the management and type of renal stones (no. 248) CT: computed tomography

Variables	No. (%)
Imaging modality used to confirm the diagnosis:	
Abdominal plain X-Ray	57 (23)
Non-contrast-enhanced CT (NCCT) scan	238 (96)
Ultrasonography (US)	26 (10.5)
Type of surgical intervention:	
Ureteroscopic/laser fragmentation	191 (77)
Retrieval/percutaneous nephrolithotomy (PCNL)	42 (16.9)
Shockwave lithotripsy (SWL)	3 (1.2)
Medical/conservative treatment	13 (5.2)
Stone location:	
Kidney	105 (42.3)
Ureter	64 (25.8)
Kidney and ureter	82 (33.1)
If the stones are in the kidney or ureter, are they:	
Bilateral	90 (36.3)
Unilateral	158 (63.7)
Number of stones identified through CT scan:	
1	88 (35.5)
2	52 (21)
3	24 (9.7)
4	15 (6)
5	11 (4.4)
More than 5 stones	58 (23.4)
Diameter of the largest stone identified:	
≤ 5 mm (0.5 cm)	60 (24.2)
6–9 mm (0.6-0.9 cm)	70 (28.2)
10–19 mm (1–1.9 cm)	89 (35.9)
≥ 20 mm (2 cm)	29 (11.7)

**Figure 1:**
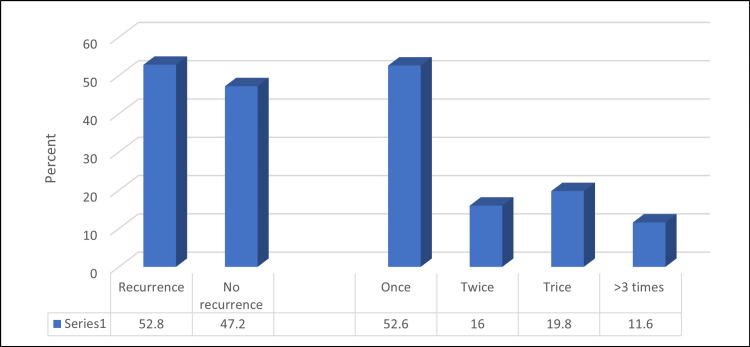
Percentage distribution of the studied patients according to recurrence (previous history of renal stone) and recurrence frequency (No. 248)

Among the studied 248 patients, 131 (52.8%) reported a previous history of renal stones. Out of these, 69 (52.6%), 21 (16%), 26 (19.8%), and 15 (11.6%) had this occur once, twice, thrice, and more, respectively (Figure 1). Table 3 demonstrates that recurrence was significantly higher among the patients who had chronic diseases (especially HTN), a stone location other than solely the kidney, unilateral stones, and a stone diameter of 10-19 mm (1-1.9 cm; p < 0.05). On the other hand, a non-significant relationship was found between the renal stones recurrence the and patients’ demographics, family history of renal stones, or other chronic diseases (p > 0.05). The relationship between the mean BMI and the clinical data related to renal stones is illustrated in Table 4, indicating a non-significant relationship between the mean BMI and all the clinical data related to renal stones (p > 0.05).

**Table 3:** Relationship between renal stone recurrence and patients' demographics, family history of renal stones, and chronic diseases (no. 248) N.B.: * = Mann-Whitney U test BMI: body mass index; DM: diabetes mellitus; HTN: hypertension; CVD: cardiovascular disease; UTI: Urinary tract infection; GIT: gastrointestinal tract; CT: computed tomography

Variables	Recurrence		χ2	P-value
	No	Yes		
	No. (%)	No. (%)		
Age	48.85 ± 13.32	49.03 ± 15.55	0.12*	0.899
BMI (kg/m^2^)	29.48 ± 6.21	29.41 ± 6.02	0.13*	0.895
Gender:			1.03	0.309
Female	41 (35)	38 (29)		
Male	76 (65)	93 (71)		
Nationality:			3.26	0.071
None-Saudi	32 (27.4)	50 (38.2)		
Saudi	85 (72.6)	81 (61.8)		
Residency:			2.88	0.09
Jeddah	85 (72.6)	107 (81.7)		
Outside Jeddah	32 (27.4)	24 (18.3)		
First/second-degree relative diagnosed with kidney stone:			1.8	0.18
No	117 (100)	129 (98.5)		
Yes	0 (0.0)	2 (1.5)		
Chronic diseases:			5	0.025
No	34 (29.1)	56 (42.7)		
Yes	83 (70.9)	75 (57.3)		
If yes, specify:				
DM	29 (24.8)	32 (24.4)	0.004	0.948
HTN	21 (17.9)	40 (30.5)	5.27	0.022
CVD	3 (2.6)	2 (1.5)	0.33	0.562
Recurrent UTI	2 (1.7)	3 (2.3)	0.1	0.754
GIT disorders	2 (1.7)	3 (2.3)	0.1	0.745
Stone location:				
Kidney:			4.83	0.028
Yes	41 (35)	64 (48.9)		
No	76 (65)	67 (51.1)		
Ureter:			0.8	0.37
Yes	34 (29.1)	30 (22.9)		
No	83 (70.9)	101 (77.1)		
Kidney and ureter:			3.56	0.059
Yes	42 (35.9)	40 (30.5)		
No	75 (64.1)	91 (69.5)		
If the stones are in the kidney or ureter, are they:			5	0.025
Bilateral	34 (29.1)	56 (42.7)		
Unilateral	83 (70.9)	75 (57.3)		
Number of stones identified through CT scan:			4.37	0.498
1	46 (39.3)	42 (32.1)		
2	26 (22.2)	26 (19.8)		
3	13 (11.1)	11 (8.4)		
4	6 (5.1)	9 (6.9)		
5	4 (3.4)	7 (5.3)		
More than 5 stones	22 (18.8)	36 (27.5)		
Diameter of the largest stone identified			13.5	0.004
≤5 mm (0.5 cm)	24 (20.5)	36 (27.5)		
6–9 mm (0.6–0.9 cm)	46 (39.3)	24 (18.3)		
10–19 mm (1–1.9 cm)	36 (30.8)	53 (40.5)		
≥ 20 mm (2 cm)	11 (9.4)	18 (13.7)		

**Table 4:** Relationship between mean BMI and clinical data related to renal stones (no. 248) N.B.: * = Mann-Whitney U test, ** = Kruskal-Wallis test CT: computed tomography

Variables	BMI (Mean ± SD)	Test	P-value
Stone location:			
Kidney		0.33*	0.741
No	29.56 ± 6.13		
Yes	29.29 ± 6.09		
Ureter:		1.04*	0.295
No	29.23 ± 6.01		
Yes	30.05 ± 6.35		
Ureter:		0.96*	0.335
No	29.65 ± 6.21		
Yes	29.02 ± 5.88		
If the stones are in the kidney or ureter, are they:		0.57*	0.563
Bilateral	29.88 ± 6.05		
Unilateral	29.2 ± 6.13		
Number of stones identified through CT scan:		5**	0.798
1	29.14 ± 6.05		
2	28.93 ± 6.42		
3	31.21 ± 7.13		
4	29.27 ± 4.13		
5	28.57 ± 6.58		
More than 5 stones	29.85 ± 5.83		
Diameter of the largest stone identified:		3**	0.377
≤ 5 mm (0.5 cm)	29.38 ± 6.06		
6–9 mm (0.6–0.9 cm)	29.67 ± 6.72		
10–19 mm (1–1.9 cm)	29.88 ± 5.81		
≥ 20 mm (2 cm)	27.64 ± 5.38		

Furthermore, a multivariate logistic regression analysis was conducted to assess the risk factors (i.e., independent predictors) of recurrence among the patients, which found that having a Saudi nationality and any chronic disease in general were risk factors (i.e., independent predictors) of recurrence among the studied patients (Table 5). The analysis also found diabetes to be protective against the recurrence of renal stones, as a high recurrence rate of renal stones was found among those who did not have diabetes.

**Table 5:** Multivariate logistic regression analysis of risk factors of recurrence among the studied patients (no. 248) BMI: body mass index; DM: diabetes mellitus; HTN: hypertension; CVD: cardiovascular disease; UTI: Urinary tract infection; GIT: gastrointestinal tract

Variables	B	Wald	P-value	Odds Ratio (OR) (CI: 95%)
Age	0.01	0.99	0.317	0.98 (0.96–1.01)
BMI (kg/m^2^)	0.01	0.247	0.619	0.98 (0.94–1.03)
Gender	0.2	0.48	0.487	1.22 (0.68–2.19)
Nationality	0.58	3.93	0.047	1.79 (1–3.18)
Residency	0.42	1.68	0.194	0.65 (0.34–1.24)
First/second-degree relative diagnosed with kidney stones	1.34	1.05	0.904	0.01 (0.3–1.21)
Chronic diseases	1.99	6.54	0.011	0.13 (0.02–0.63)
DM	1.77	7.46	0.006	5.89 (1.56–21.03)
HTN	0.16	0.11	0.736	0.83 (0.29–2.36)
CVD	1.33	1.42	0.233	2.79 (0.42–4.01)
Recurrent UTI	1.6	1.01	0.314	3.18 (0.33–3.5)
GIT disorders	0.79	0.46	0.497	2.21 (0.22–2.92)

## Discussion

Nephrolithiasis, also known as renal stones, is a common disease with more cases reported among men than women. Studies have found that having a previous history of the disease increases the recurrence of further episodes [15]. A meta-analysis of 53 articles and 488,130 enrolled patients, out of whom 17.4% experienced recurrence, found 12 demographic and clinical factors that increase the recurrence [16], including younger age, higher BMI, family history of renal stones, personal history of renal stones, hypertension, uric acid stone, being Caucasian, suspected renal stone episode before the first confirmed stone episode, the requirement for surgery, any concurrent asymptomatic (non-obstructing) stone, pelvic or lower pole kidney stone, and completion of 24-hour urine test.

As pointed out in the meta-analysis [16], there are certain inconsistencies in studies regarding the potential risk factors of recurrence. For example, although higher BMI was a risk factor for recurrent renal stones in the meta-analysis, a retrospective study involving 1496 patients [17] and a cohort study of 2,239 first-time stone formers (out of which 707 developed recurrences over a median of 11.2 years) [18] did not find an association between BMI and recurrence. Such disagreements in the studies involve other factors, such as the female gender, diabetes, and hypertension, among others; moreover, it reflects the differences in methodologies, population of the study, and the follow-up period.

In the present study, we retrospectively analyzed the demographic and clinical data of 248 patients with radiologically proven symptomatic renal stones who were managed at our hospital from November 2018 to November 2021. The primary goal of this study was to explore the association between obesity and the recurrence rate of renal stone formation. The mean age of the studied patients was 48.91 ± 14.51 years, and their mean BMI was 29.44 ± 6.1 kg/m^2^. We found the disease to be more common among men (as 68.1% of our participants were men). Moreover, 90 patients (36.3%) had chronic illnesses, and 131 (52.8%) had a previous history of renal stones. Additionally, most of the 248 patients (35.5%) had only one stone, with the majority of the stones (35.9%) being medium size (i.e., the stone diameter of 10-19 mm [1-1.9 cm]).

Consistent with the findings of the two aforementioned studies [17,18] and in disagreement with the meta-analysis [16], we did not find a relationship between obesity and the recurrence rate of renal stones. However, in the univariate analysis conducted through the chi-squared test (χ2), a significant association (p < 0.05) was found between the recurrence rate and chronic diseases (more specifically, HTN), unilateral stones, and a stone diameter of 10-19 mm (1-1.9 cm). Not many studies in the past have examined the influence of HTN on the recurrence of renal stones. Liu et al. [19] found the urine microbiome of the patients with a previous history of renal stones to be altered by HTN, which predisposed them to develop more renal stones and caused disease recurrence. Notably, pelvic or lower pole kidney stones are predisposed to more recurrence [16]. However, we found that the stones located in the kidney are protective against recurrence.

Moreover, a multivariate logistic regression analysis of the studied patients found Saudi nationality or having any chronic diseases to be risk factors (i.e., independent predictors) for recurrence. Additionally, renal stones and diabetes demonstrate a reciprocal relation [20], as having either one of the two increases the risk of acquiring the other. While we found diabetes to be protective against the recurrence of renal stones in the multivariate analysis, Iremashvili et al. [17] found it to be a potential risk factor.

Our study had certain limitations. First, the data were obtained from a single institution. Second, the sample size of the patients (N = 248, 131 patients with recurrence while 117 without) was small. Third, the study is a retrospective analysis of the information from the health records with many patients having missing demographic and clinical data. Finally, our primary goal was to study the relation between obesity and the recurrence rate of renal stones. Although no significant association was found, that cannot be definitively concluded as we were unable to fully study the metabolic syndrome. For instance, no analysis of dyslipidemia or weight circumference was conducted. Therefore, more studies are needed to test that association.

## Conclusions

Although obesity was reported to increase the risk of renal stone formation, we did not find it to be associated with an increased recurrent rate of the disease in the Saudi Arabian population, which is inconsistent with other studies conducted on this area in other countries. However, our study found Saudi nationality, chronic diseases (more specifically, HTN), unilateral stones, and a stone diameter of 10-19 mm (1-1.9 cm) risk factors for the recurrence of renal stones. Additionally, we identified diabetes and the kidney as stone location protective factors against recurrence. The sample size of the study was small and therefore, larger studies are needed to prove these findings.
